# A multi-disciplinary, comprehensive approach to management of children with heterotaxy

**DOI:** 10.1186/s13023-022-02515-2

**Published:** 2022-09-09

**Authors:** Thomas G. Saba, Gabrielle C. Geddes, Stephanie M. Ware, David N. Schidlow, Pedro J. del Nido, Nathan S. Rubalcava, Samir K. Gadepalli, Terri Stillwell, Anne Griffiths, Laura M. Bennett Murphy, Andrew T. Barber, Margaret W. Leigh, Necia Sabin, Adam J. Shapiro

**Affiliations:** 1 Department of Pediatrics, Pulmonary Division, University of Michigan Medical School, 1500 E. Medical Center Drive, Ann Arbor, MI USA; 2 Department of Medical and Molecular Genetics, Indiana University School of Medicine, Indianapolis, IN USA; 3 Department of Cardiology, Boston Children’s Hospital, Harvard Medical School, Boston, MA USA; 4 Department of Cardiac Surgery, Boston Children’s Hospital, Harvard Medical School, Boston, MA USA; 5 Department of Surgery, Section of Pediatric Surgery, University of Michigan Medical School, Ann Arbor, MI USA; 6 Department of Pediatrics, Infectious Disease Division, University of Michigan Medical School, Ann Arbor, MI USA; 7 Department of Pediatrics, Pulmonary/Critical Care Division, Children’s Minnesota and Children’s Respiratory and Critical Care Specialists, Minneapolis, MN USA; 8 Department of Pediatrics, Division of Pediatric Psychiatry and Behavioral Health, University of Utah, Primary Children’s Hospital, Salt Lake City, UT USA; 9 Department of Pediatrics, Division of Pulmonology, University of North Carolina School of Medicine, Chapel Hill, NC USA; 10 Heterotaxy Connection, Eagle Mountain, UT USA; 11 Department of Pediatrics, McGill University Health Centre Research Institute, Montreal, QC Canada

**Keywords:** Heterotaxy, Laterality disorder, Congenital heart disease, Asplenia

## Abstract

Heterotaxy (HTX) is a rare condition of abnormal thoraco-abdominal organ arrangement across the left–right axis of the body. The pathogenesis of HTX includes a derangement of the complex signaling at the left–right organizer early in embryogenesis involving motile and non-motile cilia. It can be inherited as a single-gene disorder, a phenotypic feature of a known genetic syndrome or without any clear genetic etiology. Most patients with HTX have complex cardiovascular malformations requiring surgical intervention. Surgical risks are relatively high due to several serious comorbidities often seen in patients with HTX. Asplenia or functional hyposplenism significantly increase the risk for sepsis and therefore require antimicrobial prophylaxis and immediate medical attention with fever. Intestinal rotation abnormalities are common among patients with HTX, although volvulus is rare and surgical correction carries substantial risk. While routine screening for intestinal malrotation is not recommended, providers and families should promptly address symptoms concerning for volvulus and biliary atresia, another serious morbidity more common among patients with HTX. Many patients with HTX have chronic lung disease and should be screened for primary ciliary dyskinesia, a condition of respiratory cilia impairment leading to bronchiectasis. Mental health and neurodevelopmental conditions need to be carefully considered among this population of patients living with a substantial medical burden. Optimal care of children with HTX requires a cohesive team of primary care providers and experienced subspecialists collaborating to provide compassionate, standardized and evidence-based care. In this statement, subspecialty experts experienced in HTX care and research collaborated to provide expert- and evidence-based suggestions addressing the numerous medical issues affecting children living with HTX.

**Supplementary Information:**

The online version contains supplementary material available at 10.1186/s13023-022-02515-2.

## Introduction

Heterotaxy (HTX) is a genetically complex condition of abnormal thoraco-abdominal organ arrangement across the left–right axis of the body, with an incidence of 0.81 per 10,000 births [1]. Children with HTX may have multiple organ manifestations including complex cardiovascular malformations (CVM) in over 80%, intestinal rotational abnormalities, impaired splenic function, dysmotile respiratory cilia and other abnormalities [2–4]. HTX can be caused by single gene mutations, chromosomal derangements or as part of a genetic syndrome. Long-term survival after cardiac surgery in HTX is estimated to be approximately 60% at 15 years but varies according to CVM complexity and other associated comorbidities [5, 6]. No comprehensive, evidence-based guidelines exist to direct the diagnosis, surveillance, and treatment of children with HTX. Our aim is to offer a comprehensive, multidisciplinary experience- and evidence-based approach to managing the common clinical manifestations of HTX.

## Methodology

Subspecialty experts were invited to participate if they had significant experience in HTX research and clinical care and endorsement from the Heterotaxy Connection, a non-profit, patient advocacy organization dedicated to supporting and educating patients and families with HTX. Given the paucity of vigorous clinical studies in patients with HTX, evidence-based recommendations using a GRADE approach was not possible. The management suggestions in this document are the opinion of experts with knowledge of scientific evidence and personal experience [7]. After a literature review (using Pubmed and Embase), manuscript drafts were circulated and discussed between authors by virtual conferences and email communications. After successive iterations, HTX definitions and management suggestions were unanimously approved by all authors and by the Heterotaxy Connection governing board. A summary of clinical management suggestions can be found in Table 1.

**Table 1:** Summary of management suggestions for patients with HTX

**Suggestions for every patient**
General
Patients should establish a medical home in a tertiary care center with medical subspecialty expertise (cardiology, genetics, immunology) and resources to provide comprehensive care
Primary care physicians should play a critical role in arranging routine and disease-specific immunizations, managing fevers and acute illnesses, offering medical surveillance for potential complications (midgut volvulus, biliary atresia) and coordinating care with subspecialists
Genetic workup
Patients should be evaluated by a medical geneticist for phenotyping, appropriate genetic testing and family counseling
Cardiovascular malformations
Patients should be evaluated by a pediatric cardiologist with a basic assessment of cardiac anatomy, function, and electrophysiology
Parents should be advised to seek appropriate pre-natal care, including screening fetal echocardiogram, for future pregnancies
Splenic dysfunction and Immunodeficiency
Patients should undergo an abdominal ultrasound to assess spleen presence and anatomy
Patients should be evaluated by an expert in immunology or infectious disease to help guide the assessment of spleen function, immunizations and antimicrobial prophylaxis
Patients with asplenia or functional hyposplenism should seek prompt medical attention with a fever > 38.5°Celsius (101.3°F)
Patients with asplenia or functional hyposplenism should receive all routine childhood vaccines, as well as the 23-valent pneumococcal and meningococcal vaccines in accordance with local guidelines and in consultation with an expert in immunology or infectious disease
Intestinal rotational abnormalities and biliary atresia
Families should be advised to seek immediate medical attention if patients show signs of volvulus (feeding intolerance, bilious vomiting, vague abdominal pain) or biliary atresia (jaundice, acholic stools, dark urine)
Upper gastrointestinal radiography should be performed in a child with symptoms concerning for volvulus or small bowel obstruction
A diagnosis of volvulus requires a Ladd’s procedure
Patients with concerning signs of biliary atresia should be urgently evaluated by a pediatric gastroenterologist or a pediatric surgeon
Respiratory ciliary dysfunction
Patients should be screened for clinical signs and symptoms suggestive of PCD
Patients with a chronic wet cough, chronic nasal congestion, recurrent oto-sino-pulmonary infections or unexpected post-operative pulmonary complications should be referred to a pediatric pulmonologist for evaluation of PCD and other lung diseases
Evaluation for PCD should include nasal nitric oxide testing and/or analysis of known PCD genes. If these investigations are unavailable or inconclusive, the patient should have ciliary ultrastructural analysis by electron microscopy and/or be referred to a pulmonologist at a PCD Foundation Clinical and Research Center
Psychological concerns
Providers should screen for developmental and psychological disorders annually
**Applicable on a case-by-case basis**
General
Patients should be referred to additional pediatric subspecialists based on concerns for individual system involvement (pulmonology, surgery, gastroenterology, psychology, etc.)
Genetic workup
Genome sequencing should be considered when commercial genetic panels fail to explain the clinical phenotype
Cardiovascular malformations
Cardiac imaging and functional studies, including CMR, CCT and cardiac catheterization should be considered as needed to help evaluate cardiac structure and function
Prolonged electrocardiographic (e.g. Holter) monitoring should be considered to help detect dysrhythmias not seen on a standard EKG
Splenic dysfunction and Immunodeficiency
Prophylactic antimicrobial therapy among patients with functional hyposplenism can usually be discontinued at age 5 but can be continued longer in high-risk patients
Intestinal rotational abnormalities and biliary atresia
Management of patients with complex CVM and asymptomatic IRA undergoing elective repair should be guided by a multidisciplinary team of pediatric cardiologists and surgeons
Respiratory ciliary dysfunction
Peri-operative optimization of lung function with antibiotics and increased airway clearance should be considered among patients with PCD, other forms of chronic lung disease or a history of post-operative pulmonary complications
**Things to avoid in patients with HTX**
Routine upper gastrointestinal radiography screening for intestinal malrotation among asymptomatic patients is not recommended

## Nomenclature and defintions of heterotaxy

**Fig. 1:**
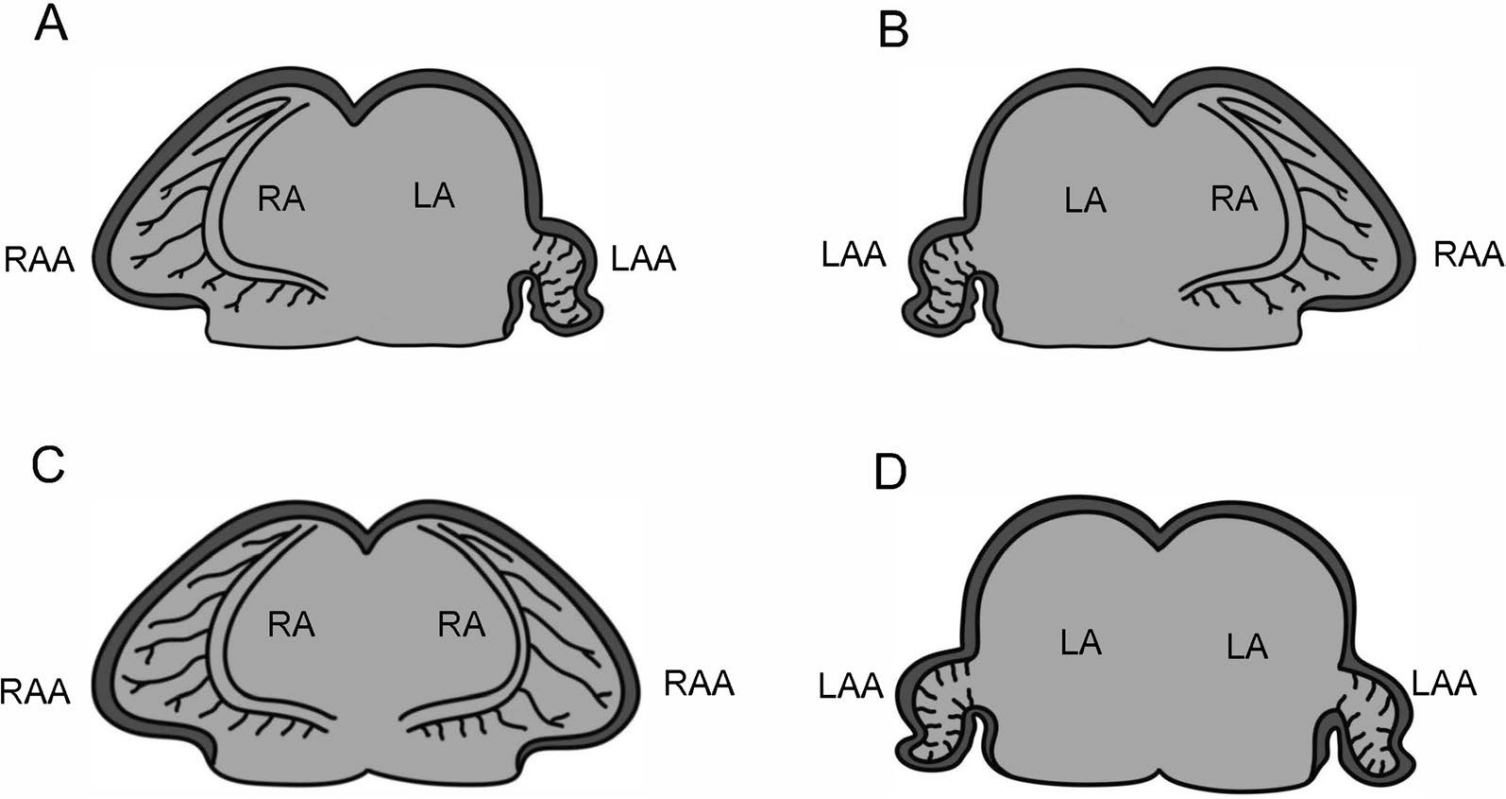
Variations of atrial appendage isomerism. Adapted from Jacobs et al. [8]. *LA* left atrium, *LAA* left atrial appendage, *RA* right atrium, *RAA* right atrial appendage. Four possible variations of right (triangular) and left (tubular) atrial appendages including the usual arrangement (**A**), mirror-imaged arrangement (**B**), right atrial appendage isomerism (**C**), and left atrial appendage isomerism (**D**)

The development of morphologically right-sided structures on one side of the body and morphologically left-sided structures on the other side is termed lateralization. Normal lateralization is termed “situs solitus”. Mirror-imaged lateralization is called “situs inversus totalis”. “Situs ambiguus” is defined as a spectrum of various organ laterality defects occurring between situs solitus and situs inversus totalis. The National Birth Defects Prevention Study (NBDPS) [1] defines HTX as abnormal symmetry of the viscera in at least three categories, including cardiac, vascular, and abdominal compartments (Table 2). Although the specific clinical definitions vary across fields, and some use the terms HTX and situs ambiguus interchangeably, HTX is broadly defined as abnormal thoraco-abdominal organ arrangement across the left–right axis of the body, usually including complex CVM. The term “isomerism” refers to a situation where morphologically right structures or morphologically left structures are found on both sides of the body (Fig. 1) [8]. Similarly, cardiac isomerism refers to duplicated atrial appendages, unique structures on the left (narrow, tubular) and right (triangular) atria. Individuals with left isomerism (also called polysplenia syndrome) frequently have bilateral left atria, two bi-lobed lungs and multiple spleens. Conversely, individuals with right isomerism (also called asplenia or Ivemark syndrome [9]), frequently have bilateral right atria, two tri-lobed lungs, asplenia, and more severe CVM. However, atrial appendages are often discordant with thoraco-abdominal sidedness [10] so lateralization of atrial appendages, liver, spleen and lungs should be assessed independently. Many studies focus specifically on cases of HTX with complex CVM (HTX-CCVM) in which the incidence of splenic and intestinal abnormalities might not truly reflect the broader population of HTX patients. In this document, to encompass the wide spectrum of HTX phenotypes seen across human disease, HTX will refer to all cases of organ laterality defects excluding situs inversus totalis, and including those with complex CVM, abdominal defects sparing the heart (e.g. situs inversus abdominalis or polysplenia with interrupted inferior vena cava), and even isolated organ defects with a disease known to affect organ laterality [e.g. polysplenia or intestinal malrotation in primary ciliary dyskinesia (PCD)].

**Table 2:** Heterotaxy diagnostic criteria from the National Birth Defects Prevention Study (modified from Lin et al. [1] and Foerster et al. [103]). Classically, the diagnosis of HTX requires three out of eight features

1. Characteristic congenital heart defect
a. Pulmonary venous anomalies (TAPVR, PAPVR)
b. Atrial anomalies (atrial situs ambiguus or inversus, common atrium)
c. Common atrioventricular canal (or septal) defects
d. Ventricular abnormalities (hypoplastic or single left ventricle, hyoplastic or single right ventricle, ventricular malposition (e.g. L-loop, superior-inferior, criss-cross))
e. Ventriculo arterial alignment abnormalities (double-outlet ventricle, D-loop TGA, L-loop TGA, truncus arteriosus, TOF (including TOF/PS, TOF/PA, and TOF/APV))
f. Ventricular outflow abnormalities (subvalvar/valvar PS, PA with intact ventricular septum, PA with ventricular septal defect (not TOF-type), valvar or subvalvar aortic stenosis, coarctation of the aorta)
2. Biliary atresia
3. Abdominal situs abnormality
a. Abdominal situs inversus
b. Midline or transverse liver
c. Midline aorta
d. Ipsilateral aorta and IVC
4. Spleen abnormality
a. Asplenia
b. Polysplenia
c. Single right-sided spleen
5. Isomerism of bronchi
a. Bilateral left bronchial morphology (bilateral hyparterial bronchus)
b. Bilateral right bronchial morphology (bilateral eparterial bronchus)
6. Isomerism of lungs
a. Bilateral two lobes (left-sidedness)
b. Bilateral three lobes (right-sidedness)
7. Similar morphology of atrial appendages (“atrial isomerism”)
8. Two of the following
a. A systemic venous anomaly
1. Bilateral SVC
2. Interrupted IVC
3. Unroofed (absent) coronary sinus
b. Intestinal malrotation (nonrotation, incomplete rotation, reverse rotation)
c. Absent gallbladder

*APV* absent pulmonary valve, *IVC* inferior vena cava, *PAPVR* partial anomalous pulmonary venous return, *PA* pulmonic atresia, *PS* pulmonic stenosis, *SVC* superior vena cava, *TAPVR* total anomalous pulmonary venous return, *TGS* transposition of the great arteries, *TOF* tetralogy of fallot

## Pathophysiology of heterotaxy

Establishment of left–right axis asymmetry in early embryogenesis is regulated by motile and non-motile (sensory) cilia. The primitive left–right organizer (LRO) is a transient structure, appearing in the third week of human gestation, and organizes establishment of laterality. The process begins with the primitive LRO sensing expression of a tightly, temporally regulated set of genes [11]. The LRO is comprised of cells arranged in a tear-drop shaped structure with pit cells projecting motile cilia surrounded by crown cells projecting non-motile cilia. Pit cell cilia lack the central apparatus doublet and radial spokes that are characteristic of most motile cilia. As a result, animal models and humans with genetic defects in radial spoke or central apparatus proteins develop motile, respiratory cilia impairment and chronic lung disease but not laterality defects [12]. A “two cilia model” hypothesis suggests that the leftward fluid flow generated by motile cilia is sensed by non-motile, mechanosensory cilia at the periphery of the LRO [13]. It is not known whether the peripheral non-motile cilia detect a chemical (Ca^2+^) or mechanical force. As a result of the coordinated clockwise ciliary motion of the pit cell cilia and subsequent leftward fluid flow, degradation of *Dand5* gene expression is increased on the left leading to increased expression of the transforming growth factor β (TGFβ) ligand NODAL at the LRO [14]; NODAL triggers activation of its own gene expression in the left lateral plate mesoderm [11, 15] while NODAL antagonists LEFTY1 and LEFTY2 restrict its activity [16]. Left-sided expression of the homeobox transcription factor, PITX2, induced by NODAL, further mediates asymmetric morphogenesis [17]. The lateral plate mesoderm signals expression of specific targets that alter cellular morphology creating the downstream effect such as cardiac looping [11]. This pathway is well conserved across model organisms. However, variants in these genes are responsible for few cases of heterotaxy in humans [18]. In addition to TGFβ signaling, developmental signaling pathways that have distinct functions in establishing the left–right axis include Wnt, planar cell polarity (PCP), and hedgehog signaling [18].

Leftward flow at the LRO requires motile cilia to be positioned posteriorly on the dome-shaped cells, a process that is dependent on non-canonical WNT signaling to effect planar cell polarity (PCP) [19, 20]. ZIC3, a zinc-finger transcription factor member of the GLI superfamily, causes X-linked HTX and congenital heart disease in humans [11, 18]. ZIC3 is critical for LRO morphogenesis [21] and is required for normal PCP-mediated movement of LRO cilia to the posterior of each cell. Additional mechanisms by which ZIC3 causes heterotaxy remain incompletely understood but include Wnt, hedgehog, and TGFβ signaling [22].

The developing heart is the earliest organ to express laterality, explaining the common association between HTX and congenital heart disease [18, 23]. However, HTX is a systemic disorder, and can affect nearly every organ and system in the body. Children with HTX, therefore, should establish longitudinal care with a primary care provider as well as a medical home in a tertiary care facility for subspecialty and expert consultation.

## Genetics of heterotaxy

Many of the genes known to cause HTX were identified based on an understanding of the developmental pathways related to left–right patterning. HTX most commonly occurs as a sporadic condition where the genetic cause(s) is not identified [24]. Whole exome sequencing among 323 individuals with laterality defects identified known genetic variants in only 7% of subjects [24]. Although less frequently inherited as a Mendelian trait, HTX can occur as an autosomal dominant, autosomal recessive, or X-linked condition [11, 24] (Table 3). *ZIC3*, a cause of X-linked HTX, was the first gene associated with HTX but likely accounts for less than 3–5% of HTX cases in males [25, 26]. However, genetic testing that includes *ZIC3* is important for providing recurrence risk information to families. Autosomal dominant etiologies typically involve genes encoding major regulators of laterality such as TGFβ members of the NODAL signal transduction pathway [11, 24], and typically demonstrate both reduced penetrance and variable expressivity. Most autosomal recessive etiologies of HTX are genes encoding proteins important for cilia structure or function and include syndromic non-motile ciliopathies and motile ciliopathies (e.g., PCD) [11, 24, 27].

**Table 3:** Genes associated with heterotaxy in humans

Autosomal dominant	*ACVR2B, CFC1, CRELD1, FOXH1, LEFTY1, LEFTY2, NKX2.5, NODAL*
Autosomal recessive	*CFAP53, GDF1, MMP21, MNS1, PKD1L1*
Autosomal recessive (Primary ciliary dyskinesia)	*ARMC4, CCDC103, CCDC114, CCDC151, CCDC39, CCDC40, CCDC65, CCNO, CFAP298, CFAP300, DNAAF1, DNAAF2, DNAAF3, DNAAF4, DNAAF5, DNAH1, DNAH11, DNAH5, DNAH9, DNAI1, DNAI2, DNAJB13, DNAL1, DRC1, FOXJ1, GAS2L2, GAS8, HYDIN, LRRC56, LRRC6, MCIDAS* *NEK10, NME8, PIH1D3, RSPH1, RSPH3, RSPH4A, RSPH9, SPAG1, STK36, TP73, TTC12, TTC25, ZMYND10*
X-Linked	*ZIC3*

**Fig. 2:**
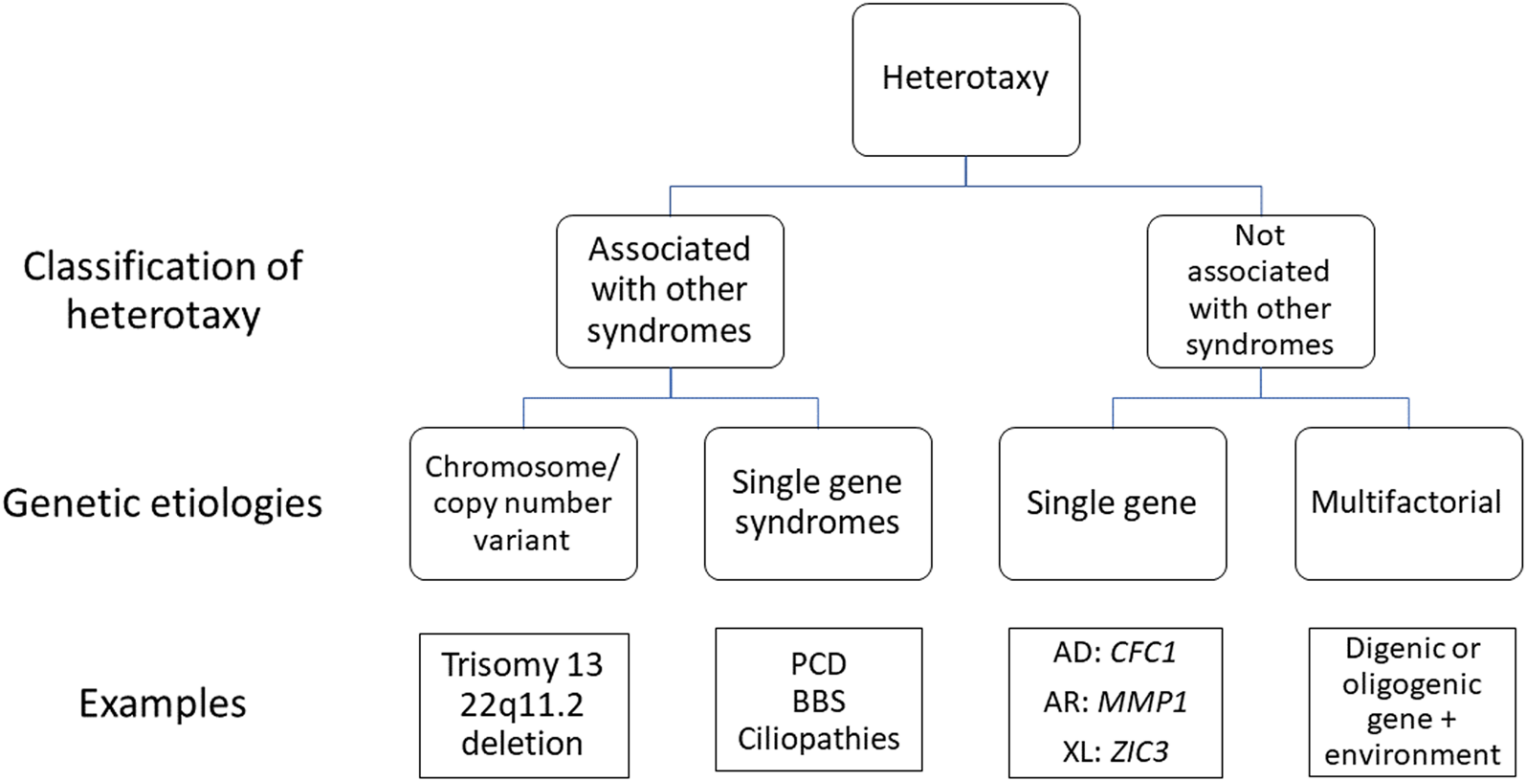
Classification of genetic etiologies of heterotaxy. *AD* autosomal dominant, *AR* autosomal recessive, *BBS* Bardet Biedl syndrome, *PCD* primary ciliary dyskinesia, *XL* X-linked

HTX can be a phenotypic feature in a number of other genetic syndromes (Table 4, Fig. 2). In addition to their non-random association with HTX, these syndromes have a variety of abnormalities not typical for HTX including subtle organ laterality defects, dysmorphic features, congenital anomalies, neurodevelopmental anomalies and predispositions for endocrinologic abnormalities (see Additional file 1). Syndromes involving dysfunction of motile and non-motile cilia, or ciliopathies, are the most numerous and best understood of these syndromes [28]. The vast developmental, sensory and mechanical functions of cilia help explain the heterogeneous and multi-organ manifestations of ciliopathies [11, 23, 28].

**Table 4:** Known genetic syndromes with heterotaxy in the disease phenotype

22q11.2 Deletion Syndrome
Aglossia with Situs Inversus
Agnathia-Otocephaly Complex [104]
Bardet-Biedl Syndrome [105]
Cardiac Urogenital Syndrome [106]
Cardiofacioneurodevelopmental Syndrome [107]
Carpenter Syndrome [108]
DK Phocomelia Syndrome
Ellis-Van Creveld Syndrome [109]
Galactosialidosis
Hennekam Lymphangiectasia-Lymphedema Syndrome [110]
Johanssen-Blizzard Syndrome
Marden-Walker Syndrome
Nephronophthisis
Oculo-Auriculo-Vertebral Spectrum Disorder
Polycystic Kidney Disease
Primary Ciliary Dyskinesia
Renal-Hepatic-Pancreatic Dysplasia
Sandestig-Stefanova Syndrome
Short Rib Thoracic Dysplasia 3

There are no formal guidelines for genetic evaluation or testing for patients with HTX. Evaluation by a geneticist is recommended in all HTX patients to help characterize the phenotype, identify syndromic ciliopathies and order appropriate testing. Identification of a syndrome can help guide management and genetic counseling. Patients with HTX should undergo molecular evaluation for copy number and sequence variants [27]. A number of commercial clinical genetic panels, using next generation sequencing, are available to investigate HTX, laterality disorders, congenital heart defects, primary ciliary dyskinesia, non-motile ciliopathies, situs inversus, and other clinical concerns. The available options for clinical genetic testing with next generation sequencing change rapidly, and genome sequencing may be considered when genetic panels fail to adequately explain the patient phenotype. Chromosome analysis/karyotype analysis is not necessary unless there is a suspicion for trisomy 13.

## Congential cardiovascular malformations

### Cardiac anatomy

Every child with HTX should be evaluated by a pediatric cardiologist at the time of diagnosis. If a CVM is identified, families should seek appropriate pre-natal care to assess for CVM in future pregnancies. Cardiac anatomy in HTX ranges from complex single ventricle CVM to a normal or nearly normal biventricular circulation [1]. Certain CVMs are more likely to be encountered in right atrial isomerism than in left atrial isomerism (Table 5), although there is tremendous variability and overlap among the two groups. The need for medical, catheter-based, or surgical therapy depends on the underlying anatomy, and a substantial proportion of morbidity and mortality among HTX patients is from cardiac disease [6].

**Table 5:** Cardiac characteristics frequently encountered in heterotaxy subtypes of left and right atrial isomerism*

Left atrial isomerism/polysplenia	Right atrial isomerism/asplenia
*Anatomic*	*Anatomic*
Interrupted inferior vena cava	Intact inferior vena cava
Normal or ipsilateral PVR, usually unobstructed	Total anomalous PVR, possibly obstructed
Two ventricles	Functionally single ventricle
Two separate AV valves	AV canal defects with a common AV valve
Normally related great arteries	Transposed or malposed great arteries
Systemic outflow obstruction	Pulmonary outflow obstruction
*Conduction*	*Conduction*
Absent sinus node	Dual sinus and AV nodes
Complete heart block	Supraventricular tachycardia

*AV* atrioventricular, *PVR* pulmonary venous return

*Table lists cardiac characteristics more likely to be encountered in each heterotaxy subtype, but there is substantial overlap between categories

Cardiac anatomic assessment requires a detailed examination of thoraco-abdominal situs, cardiac position, atrial situs, ventricular looping, atrioventricular alignments and connections, ventricular-arterial alignments, infundibular anatomy, relationships between the semilunar valves and great arteries, septal defects, and anomalies of venous return and/or arterial exit, and atrial and ventricular septal defects [29, 30]. The myocardial architecture should be assessed for non-compaction, which may predispose toward early or late cardiomyopathy [31]. Finally, patients should undergo a thorough assessment of the cardiac conduction system.

### Cardiac diagnostic imaging

Diagnostic imaging needs vary widely depending on CVM complexity and patient age. HTX is often identified in the prenatal period on screening obstetric ultrasound. This is usually followed by a detailed fetal echocardiogram [32, 33]. Information from fetal echocardiography can be used [34] to provide detailed prenatal counseling [8] and to guide perinatal resuscitation and care [35, 36].

Routine transthoracic echocardiography is the mainstay of cardiac imaging in HTX. Transesophageal echocardiography is typically reserved for perioperative guidance or for detailed valve assessment. Cardiovascular magnetic resonance imaging (CMR) permits quantification of ventricular volumes, function and flow, and a detailed characterization of anatomy in three dimensions. CMR is particularly useful in HTX to evaluate complex intracardiac anatomy prior to cardiac surgery and to assess older individuals in whom echocardiographic windows are often poor. However, CMR is limited by the need for general anesthesia in younger patients, potential renal injury from gadolinium-containing contrast agents and incompatibility with a pacemaker [37]. Cardiac computed tomography (CCT) permits rapid-acquisition and high-quality delineation of vascular anatomy with modest doses of both radiation and iodine-containing contrast [38–40]. Functional CCT is an alternative to CMR for patients with a pacemaker but requires higher doses of radiation than static CCT.

### Cardiac catheterization

Cardiac catheterization remains the primary method of hemodynamic assessment in HTX, which can be critically important for a complete physiologic understanding of a patient, most notably in pre-surgical planning for cardiac intervention. It also has an important therapeutic role in addressing numerous hemodynamic lesions and can sometimes obviate the need for surgery [41].

### Electrophysiologic assessment

Patients with HTX should undergo a thorough assessment of their conduction system. An electrocardiogram is required at baseline to characterize each patient’s native conduction and as needed when arrhythmia is suspected. In addition, strong consideration should be given to a period of prolonged electrophysiologic monitoring (e.g. 24-h or longer Holter monitor) to identify any subtle conduction abnormalities or predisposition to pathologic arrhythmia not detectable on a 10-s electrocardiogram. For patients with complex arrhythmias, catheterization-based electrophysiologic study and intervention may be required. Of note, intraoperative electrophysiologic mapping at the time of complex intracardiac repair is increasingly utilized to avoid damage to the conduction system, which is often abnormal in HTX [42].

### Cardiac surgical interventions

Cardiac surgery in early infancy is mostly guided by the severity of symptoms; surgery in older children is usually directed at establishing stable single- or two-ventricle physiology. The most common indications for surgical or catheter intervention in early infancy are obstructed total anomalous pulmonary venous return (TAPVR) and severe cyanosis due to pulmonary valvar or sub-valvar stenosis (often associated with right isomerism). Urgent surgical intervention for obstructed TAPVR in HTX carries a mortality rate of 38% [43]. The 1-year mortality after neonatal pulmonary atresia or pulmonary stenosis repair in HTX is over 12% [44]. Some neonates with polysplenia and complete heart block require urgent postnatal pacemaker placement [32].

Surgical intervention after the neonatal period involves either a single-ventricle or two-ventricle strategy. In over 50% of children with HTX, either because of severe hypoplasia of one ventricle or complex AV valve anatomy, a staged single ventricle strategy is pursued [6]. This usually starts at 3–6 months of age with a partial cavo-pulmonary (Glenn) connection in which the superior vena cava (SVC, single or bilateral) is connected directly to the corresponding branch pulmonary artery leading to passive flow of upper body venous blood into the pulmonary circulation. In patients with interrupted inferior vena cava (IVC), the azygos vein brings venous blood from the lower body into the SVC, increasing the passive blood flow into the pulmonary circulation. The second stage of single ventricle management (Fontan operation) is usually done by 2 or 3 years of age and involves connecting the inferior vena cava (IVC) to the corresponding branch pulmonary artery, completing the total cavo-pulmonary connection. Single-institution survival among children with HTX who undergo a Fontan operation is reported as 100%, 85% and 73% at 1, 10 and 15 years, respectively [45]. Immediate post Fontan mortality was found to be higher in children with HTX and CVM compared to children with similar CVM without HTX although long term mortality is similar and overall surgical outcomes have been improving [5, 46–48]. One-year survival after heart transplant was found to be lower among children with HTX (77.2%) compared to children without HTX (85.1%) although survival has been improving over time [49].

Two-ventricle repair has traditionally been reserved for children with two well-developed ventricles and two separate inlet valves. Staged ventricular recruitment, via ASD restriction without VSD closure, has been shown to help grow mildly hypoplastic ventricles and facilitate eventual two-ventricular conversion [50].

Associations between pulmonary disease, including genetically verified PCD, and cardiac surgery outcomes among children with HTX is understudied. One study of twenty-seven patients identified more post operative respiratory complications but no difference in hospital length-of-stay or mortality among patients with HTX and ciliary dysfunction when compared to patients with HTX but no ciliary dysfunction. There was a trend towards increased mortality among patients less than 10 years of age, which is when many important cardiac surgeries occur [51, 52]. Importantly, none of these patients had proven PCD through accepted diagnostic testing. Post-surgical outcomes have not been studied among HTX patients with genetically-verified PCD. Pre-existing PCD should not preclude surgery for CVM, but it should be considered when deciding the surgical strategy. Patients with chronic lung disease are known to have elevated pulmonary vascular resistance. Single-ventricle palliation requires low pulmonary vascular resistance for passive pulmonary blood flow through cavopulmonary connections and might therefore be less favorable than two-ventricle repair among those with appropriate anatomy. Careful peri-operative planning and consultation with various subspecialists, including a pulmonologist, for effective pulmonary toilet if respiratory ciliary dysfunction is proven or suspected, is critical in order to address comorbidities and optimize post-surgical outcomes.

## Splenic dysfunction and immunodeficiency

The spleen plays an important role in the innate and adaptive immune systems’ response to infection. The sinusoids within the spleen act as a mechanical filter, clearing out particulates, including bacteria. Macrophages within the splenic red pulp eradicate bacteria via phagocytosis and invoke a cytokine response to activate other leukocytes to fight infection. The splenic white pulp contains the majority of an individual’s memory B cells, and individuals with asplenia can have lower numbers of IgM memory B lymphocytes [53]. Memory B cells play a significant role in adaptive immunity, particularly in the creation of antibodies against polysaccharides found on encapsulated organisms. The spleen, therefore, plays a critical role in defending against encapsulated organisms (*Streptococcus pneumoniae*, *Haemophilus influenzae* and others) [54, 55]. Splenic anomalies are found in up to 90% patients with HTX and CVM [4], ranging from complete absence of a spleen (asplenia) to multiple spleens (polysplenia). Any variations in splenic structure may result in impaired spleen function (functional hyposplenism). Among a group of 38 patients with HTX with CVM and proven bacteremia, 79% had asplenia, but 21% had either polysplenia or anatomically normal spleens [56].

### Risk for infection in patients with HTX

Patients with anatomic asplenia or functional hyposplenism are at increased risk for infections caused by encapsulated organisms, with an annual infection rate of 4.2% [57]. Infection-related mortality is high in both the asplenic and polysplenic HTX populations [56]. However, children with anatomic asplenia have a higher incidence of infections requiring hospitalization (50%) compared to those with polysplenia or anatomically normal spleens (8%) [55]. Functional hyposplenism, therefore, is a concern even among those with multiple spleens or a normal appearing spleen and HTX. Individuals with anatomic asplenia or functional hyposplenism due to other causes (sickle cell disease, surgical splenectomy, post-traumatic functional asplenia, etc.) are estimated to have a 5% lifetime risk of bacteremia, with a mortality risk up to 600 times that of the general population [56].

### Assessment of spleen function

Assessment of Howell Jolly bodies, basophilic DNA remnants from the nucleus of erythrocyte precursor cells, under light microscopy or flow cytometry, is a widely used and sensitive way of determining splenic function [58]. Erythrocytes containing Howell Jolly bodies are usually cleared from circulation by the spleen; therefore, presence of Howell Jolly bodies suggests decreased spleen function. However, Howell Jolly body detection is unreliable before the age of two years and can miss mild forms of hyposplenism [56].

More advanced splenic function testing includes quantification of pitted erythrocytes and ^99m^Technetium-labeled scintigraphy. Erythrocytes of patients with hyposplenism contain large vacuoles of wasted materials beneath the plasma membrane. Under interference phase microscopy, the erythrocytes appear to contain “pits” [59]. Pit counts above 4% are considered indicative of hyposplenism [56]. This method is used less often as it requires specialized training and equipment [56, 60, 61]. Finally, ^99m^Technetium-labeled scintigraphy uses radionuclide tagged erythrocytes to determine the number of abnormal erythrocytes not being cleared by the spleen. An alternative approach uses TC-labelled sulphur colloids to compare the phagocytic function of the liver and spleen. Although this is a highly sensitive and specific way to detect hyposplenism, this method employs radiation, and requires more labor, expertise, and expense than more traditional methods [56, 60]. The sensitivity and specificity of ^99m^Technetium-labeled scintigraphy to detect hyposplenism in young children is not known.

Although children with anatomic asplenia can be empirically assumed to have functional hypoplenism, there is insufficient evidence to support a standardized approach to investigating hyposplenism among all children with HTX who have polysplenia or anatomically normal spleens. Therefore, splenic function in HTX should be carefully evaluated at the time of diagnosis in consultation with an expert in immunology or infectious diseases. Use of scintigraphy, pitted erythrocytes or Howell Jolly body assessment should be individualized to the patient and practices of local expert consultants. Patients with HTX should be presumed to have functional hyposplenism and treated with prophylactic antibiotics until such an assessment can be made.

### Prevention of infection

Individuals with hyposplenism should receive all routine childhood vaccines according to standard vaccination guidelines. In the United States, the Centers for Disease Control and Prevention (CDC) and Advisory Committee on Immunization Practices (ACIP) recommendations include pneumococcal (PCV13) and *H. influenzae* type b vaccines as part of the routine childhood vaccine schedule. Once the primary pneumococcal series is complete, individuals with HTX should also receive the pneumococcal polysaccharide vaccine (PPSV23) when they reach 2 years of age, and a subsequent booster dose 5 years later. The meningococcal conjugate vaccination is recommended at age 11–12 with a booster at 16. The serogroup B meningococcal vaccination is recommended for children 10 years or older. Some meningococcal vaccines can be given in infancy to children with HTX at increased risk for meningococcal disease. Careful review of national vaccine guidelines in consultation with an immunologist or infectious disease specialist is recommended to ensure a timely and comprehensive immunization schedule. The CDC offers specific recommendations for individuals with asplenia [62].

In addition to immunizations, individuals with anatomic asplenia or functional hyposplenism should be placed on daily antimicrobial prophylaxis with penicillin V (125 mg if < 3 years old; 250 mg if ≥ 3 years old) twice daily or amoxicillin 20 mg/kg/day divided twice daily to prevent infections from encapsulated organisms, including pneumococcal infections [63, 64]. Erythromycin (125 mg if < 3 years old; 250 mg if ≥ 3 years old) twice daily is a potential alternative for children with a penicillin allergy. In some patient populations, daily prophylaxis has been shown to decrease invasive pneumococcal infections by 50–80%. The National Heart, Lung and Blood Institute recommendation among children with sickle cell disease (who often develop functional hyposplenism) is to stop prophylaxis at age 5 unless the child has had a splenectomy or a history of invasive pneumococcal infection [56, 61, 63–66]. Prophylaxis can likely be safely discontinued at 5 years of age if the individual has received all appropriate vaccines, has not had a history of severe infection, and has rapid access to medical care should concerns arise [61, 64]. Consultation with an immunologist or infectious disease specialist is recommended to help guide antimicrobial prophylaxis.

### Management of fever

**Fig. 3:**
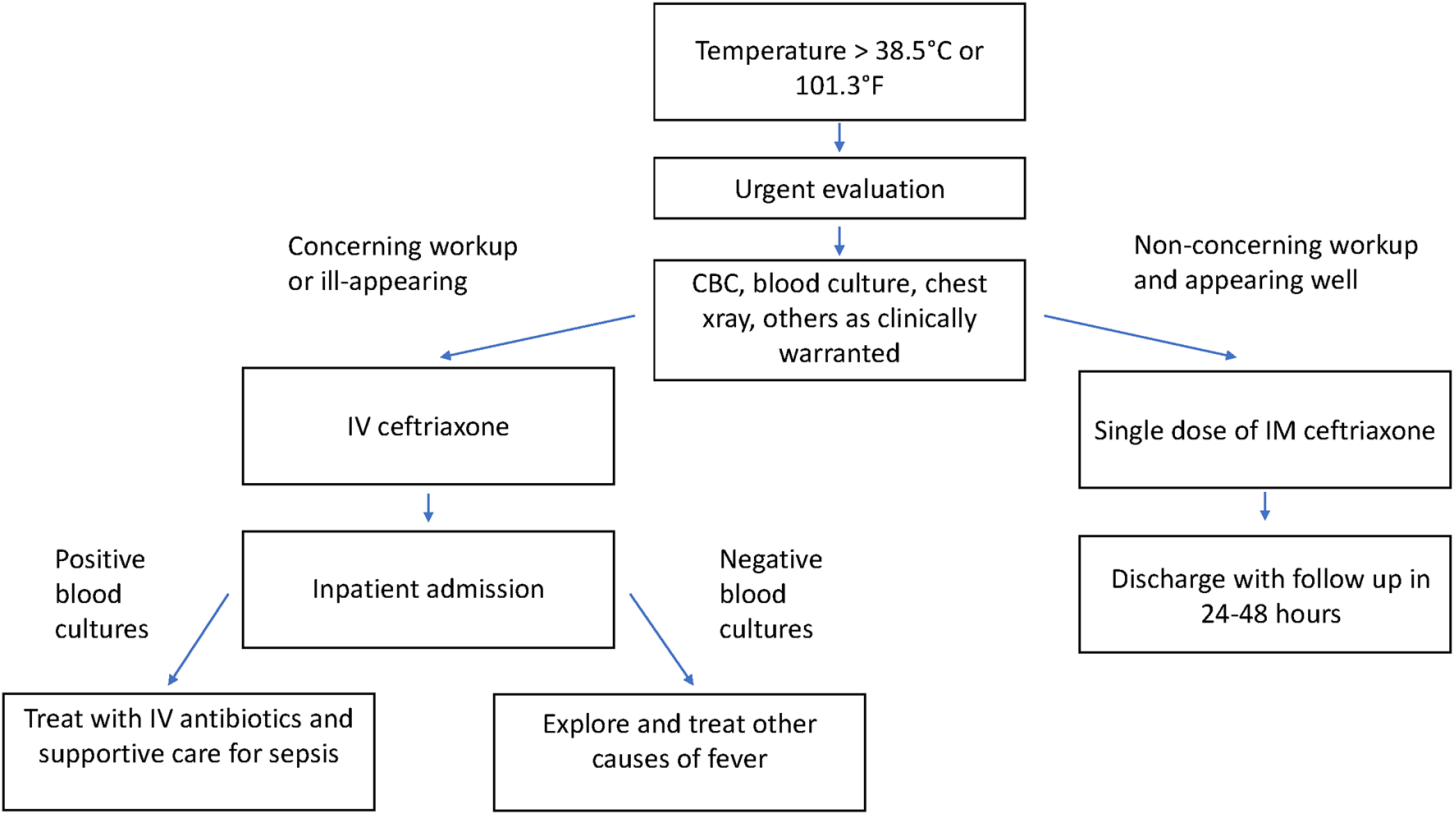
Algorithm for management of fever in a patient with asplenia or functional hyposplenism. Modified from Loomba et al. [56]. *IV* intravenous, *IM* intramuscular

Fever can be a sign of serious infection in individuals with anatomic asplenia or functional hyposplenism. Pediatric HTX patients with asplenia or functional hyposplenism who develop a temperature greater than 38.5 °C or 101.3 °F should seek immediate medical attention for prompt assessment for sepsis and treatment with empiric antibiotics. Patients with concerning bloodwork or who are ill-appearing should be admitted to the hospital; those appearing well with reliable follow up can be discharged and re-assessed in 24–48 h (Fig. 3). In a large retrospective study among patients with sickle cell disease, bacteremia was very rare in settings of temperatures less than or equal to 38.5 °C [67]. Individuals living remotely without ready access to medical care should have an emergency supply of antibiotics at home [63].

## Intestinal rotational abnormalities and biliary atresia

**Fig. 4:**
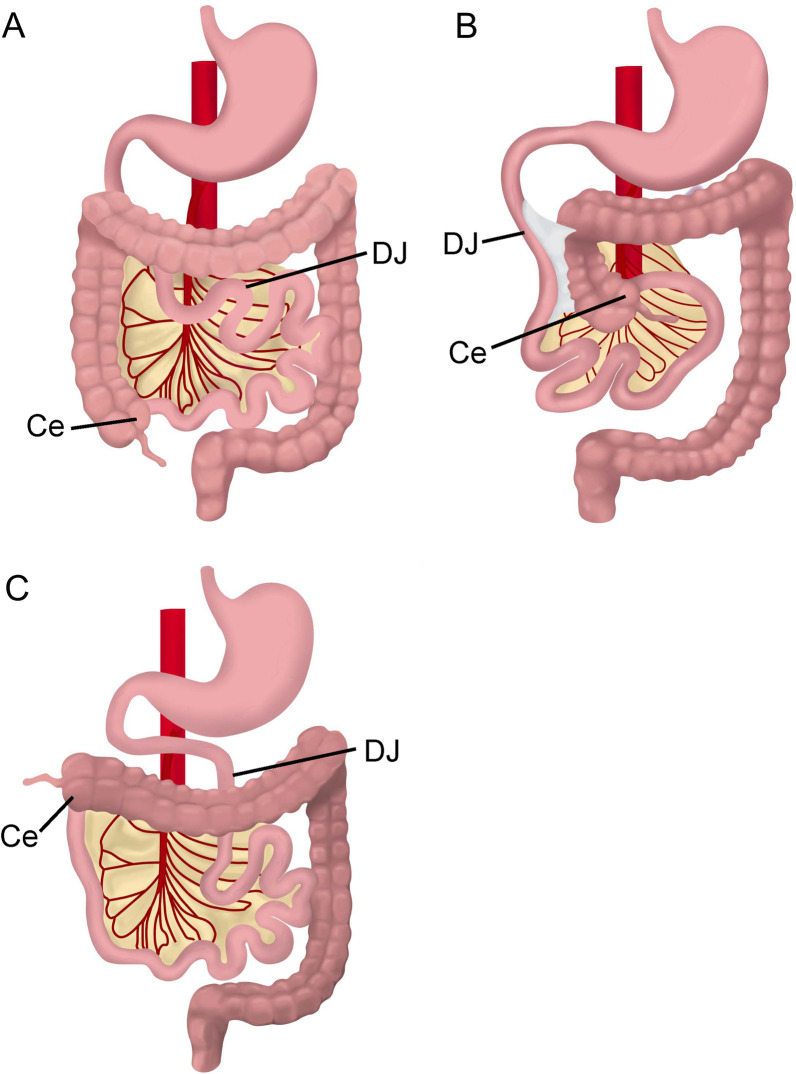
Variations of intestinal rotation. Three variations of intestinal rotation including normal fixation of the duodenojejunal junction (DJ) in the left upper quadrant and cecum (Ce) in the right lower quadrant (**A**), complete malrotation with duodenojejunal junction and cecum in close proximity leading to a narrow mesentery (**B**) and heterotaxy in which the location of the duodenojenunal junction and cecum is not normal but the mesentery is not as narrow as complete malrotation (**C**)

Intestinal rotational abnormalities (IRA), such as malrotation, are congenital conditions that might lead to twisting of the gastrointestinal tract (volvulus) causing bowel obstruction and vascular compromise. The reported incidence of malrotation among HTX patients screened for IRA is 58–72% [2, 68, 69]. Under normal conditions, the intestines undergo a predictable 270-degree counterclockwise rotation around the superior mesenteric artery (SMA). The duodenojejunal junction then anchors to the retroperitoneum in the left upper quadrant with the ligament of Treitz and the cecum anchors to the retroperitoneum in the right lower quadrant (Fig. 4A). A broad mesentery between these distant fixation points supports the superior mesenteric artery. Under classic conditions of malrotation, where the duodenojejunal junction and cecum are fixed to the retroperitoneum at abnormal points, a narrow mesentery base is formed and creates an opportunity for volvulus and interruption of SMA perfusion (Fig. 4B). However, the distance between fixation points and mesentery breadth is variable with HTX, leading to a lower risk for volvulus as compared to malrotation in patients without HTX (Fig. 4C). Symptomatic patients must undergo emergent surgery (Ladd’s procedure) to prevent bowel necrosis. Surgical risks are complicated by the additional comorbidities of HTX including CVM. There is a 3–15% lifetime risk of bowel obstruction after a Ladd’s procedure secondary to the development of new adhesions [70].

### Screening for IRA

Screening for IRAs among asymptomatic patients with HTX remains controversial [2, 68]. Although relatively higher mortality has been reported when Ladd’s procedure is delayed until development of acute volvulus symptoms, [2, 71] the surgery itself carries its own substantial risks. The rate of 30-day post-operative complications, including small bowel obstruction from adhesions, kidney failure, respiratory arrest and sepsis, ranges from 14 to 57% [2, 70–73]. Mortality of a Ladd’s procedure (13–22%) is higher in HTX patients compared to non- HTX patients, due to cardiovascular complications unique to HTX patients [71–73]. Despite the high rate of IRA associated with HTX, the overall incidence of volvulus is very low compared to the incidence in non-HTX populations. Among non-screened patients, the risk for volvulus with HTX is lower (5.8%) [2] compared to a broader population of non-HTX infants (37%), children (22%) and adults (12%) with intestinal malrotation [74]. This is likely due to the broader mesentery and variable retroperitoneal fixation points among HTX patients compared to the classic form of non-HTX intestinal malrotation with a narrow mesentery base. Given the relatively low risk for volvulus and the high rate of post-operative complications and mortality in HTX patients with CVM, routine screening and prophylactic Ladd’s procedure is not recommended in HTX [75, 76]. Families should be educated on the signs and symptoms of volvulus including feeding intolerance, bilious vomiting, and vague abdominal pain and seek prompt medical attention if these symptoms develop. Upper gastrointestinal (UGI) study is recommended to confirm volvulus, with a reported sensitivity of 96% [77].

### Surgical indications

A Ladd’s procedure, in which mesenteric bands are divided, the colon is placed on the left and the small bowel on the right, is the traditional approach to correcting an IRA. A laparoscopic approach offers low rate of recurrence [78] but might also prolong ischemia time. Once a child is diagnosed with volvulus on an UGI, Ladd’s procedure should be arranged emergently. A Ladd’s procedure in an asymptomatic patient with IRA might be considered in situations where rapid access to emergent care is not possible, including those living in remote communities. If elective surgical intervention is pursued, it should be delayed until after cavopulmonary connection or complete cardiac repair at a center with experience in pediatric cardiac anesthesia [76]. Delaying a Ladd’s procedure until establishment of a more balanced cardiovascular circulation is associated with a shorter duration of mechanical ventilation, parenteral nutrition, and length of stay without impacting hospital mortality [79].

### Biliary atresia

Although the incidence of extrahepatic biliary atresia in HTX is poorly studied, it has been reported in approximately 6% of children with HTX and CVM, which is 1000 times higher than the general population risk (0.5–0.8 per 10,000) [4, 80, 81]. Biliary atresia is the most common cause of liver transplantation and death from liver disease in children [82]. Delayed diagnosis and surgical intervention after age 3 months significantly worsens outcomes [83]. Although there are promising newborn screening programs using stool color cards or bilirubin levels, no systematic screening program exists in the United States [84, 85]. The sensitivity of routine abdominal ultrasonography is also poor, making it an unreliable screening tool for biliary atresia [86]. Therefore, families of children with HTX should be carefully educated regarding signs and symptoms suggestive of biliary atresia in the first 8 weeks of life (jaundice, acholic stools and dark urine), and children should undergo diagnostic testing under the guidance of a pediatric gastroenterologist or surgeon when symptoms appear.

## Respiratory ciliary impairment

Primary ciliary dyskinesia is a rare disorder of mucociliary impairment causing chronic oto-sino-pulmonary infections and bronchiectasis (irreversible airway dilatation due to chronic infection). Nearly 50% of patients with PCD have lateralization defects including 9–25% with HTX [3, 87, 88]. The converse—the prevalence of PCD or a milder form of ciliary impairment among patients with HTX—remains unknown. In a web-based survey of patients with HTX, 37% of respondents reported findings of bronchiectasis, chronic respiratory symptoms, or past detection of PCD-associated respiratory pathogens [89]. Among a group of 43 patients with HTX, 42% were found to have subjectively abnormal ciliary beat patterns on high-speed video microscopy analysis and abnormal nasal nitric oxide (nNO) levels either below or near the PCD diagnostic cutoff value of 77 nL/min [90]. However, none of the HTX patients in this study had genetically verified PCD.

Pulmonology consultation is recommended for children with HTX and chronic cough, nasal congestion, recurrent oto-sino-pulmonary symptoms or post-operative pulmonary complications in order to evaluate for PCD and other lung diseases [91, 92]. The diagnosis of PCD requires ≥ 2 of 4 key clinical features (unexplained neonatal respiratory distress, year-round wet cough, year-round nasal congestion, or organ laterality defects) and confirmatory diagnostic investigations including repeatedly low nasal nitric oxide (nNO) levels (< 77 nL/min), genetic testing or transmission electron microscopy (TEM) analysis of the ciliary ultrastructure [93]. However, genetic testing and TEM are negative in up to 30% of patients with PCD, and nNO measurement cannot be reliably performed before 2 years of age and may be low in other respiratory conditions. Given the technical challenges and imperfect sensitivity and specificity of these diagnostic tests, a diagnosis of PCD in a patient with an appropriate clinical history requires expert consultation unless a definitive diagnosis can be made through clinical genetic testing [93]. If nNO or genetics are unavailable or inconclusive, the patient should have ciliary ultrastructural analysis by electron microscopy and/or be referred to a pulmonologist at a PCD Foundation Clinical and Research Center.

Treatment of PCD includes regular airway clearance, aggressive and proactive antimicrobial treatment, routine supplemental vaccinations specific for respiratory illness, and careful attention to chronic ear disease and rhinosinusitis [93]. Further testing for respiratory manifestations in HTX might include pulmonary function testing, chest CT imaging for assessment of bronchiectasis secondary to chronic pulmonary infections, and bronchoscopy to assess airway caliber and anatomy with bronchoalveolar lavage or expectorated sputum for cultures to guide antimicrobial therapy. Peri-operative optimization of lung function with antibiotics and airway clearance in consultation with a pulmonologist is recommended for those with PCD, other forms of chronic lung disease or a history of post-operative pulmonary complications.

## Psychological manifestations

There are limited data describing the psychological implications of HTX. Children with a history of prolonged or repeated hospital stays, significant cardiac disease, multiple surgical interventions or repeated exposure to anesthesia, intensive care unit stays, delirium, and hypoxic events are at highest risk for neuropsychological and psychological sequelae [66, 94, 95].

### Developmental and Neuropsychological assessment

Children and adolescents with HTX may be at increased risk for developmental delays and neuropsychological sequelae, including attention deficit disorders, learning disabilities, and poor executive functioning [96]. Children with a genetic syndrome associated with HTX such as 22q11.2 Deletion Syndrome have neurodevelopmental outcomes typical for the specific syndrome. For children with HTX and CVM, neuropsychological assessment with a pediatric neuropsychologist should follow established guidelines [97]. For children with HTX without significant cardiac involvement, routine developmental screening by primary or subspecialty medical providers is warranted with a low threshold for referral to a pediatric neuropsychologist when concerns arise.

### Psychological screening, surveillance, and assessment

Medically complex children and adolescents are more likely to experience anxiety disorders, mood disorders, functional disability, and impaired social relationships than healthy peers [98]. In addition, patients with HTX may be more vulnerable to feeding and eating disorders in the context of gastrointestinal dysfunction, poor motility, chronic pain, or early disturbance of feeding (often due to cardiac and pulmonary disease). The presence of psychological disorders contributes to worse health outcomes, greater functional impairment, and poor adherence to medical regimens [99]. Therefore, primary care providers and subspecialists should screen for psychological conditions annually. In the case of positive screens, referral to medical psychologists or mental health providers experienced with children with complex medical needs is warranted. Empirically validated screening measures can be found through healthmeasures.net (the PROMIS Scales), the American Academy of Pediatrics, and the American Academy of Child and Adolescent Psychiatry. Annual psychology screening should include both parent and patient symptom reports, once developmentally appropriate.

### Central nervous system (CNS) manifestations of HTX

Congenital brain malformations were found in 46% of postmortem specimens from children with right atrial isomerism [100]. No brain malformations were found in patients with left atrial isomerism. Brain anomalies are thought to be secondary to impaired motile and non-motile ciliary function in the CNS. Indeed, hydrocephalus and other brain malformations have been reported in patients with PCD [101] and other ciliopathies [28, 102]. In the absence of a clear understanding of the clinical relevance of these abnormalities, screening is not recommended. Rather, investigations should be sought if clinical concerns for CNS abnormalities arise.

## Conclusion

Heterotaxy is a genetically complex disorder affecting multiple organ systems. Common manifestations include CVM, functional hyposplenism with immunodeficiency, intestinal rotational abnormalities, respiratory ciliary dysfunction, developmental and neuropsychological disorders, and others. Optimal care of children with HTX requires a team of expert subspecialty providers. Family and health care provider education regarding the multiple comorbidities of this disorder, and national clinical and research collaborations are needed to advance our understanding of the pathophysiology of this disorder, develop practice guidelines, and offer HTX patients the best chance at improving clinical outcomes and quality of life.

## Supplementary Information

**Additional file 1:** Known genetic syndromes with heterotaxy in the phenotype.

## Data Availability

Data sharing is not applicable to this article as no datasets were generated or analyzed during the current study.
