# Supplementary material for: A multi-disciplinary, comprehensive approach to management of children with heterotaxy
Source: Orphanet J Rare Dis. 2022 Sep 9;17:351. doi: 10.1186/s13023-022-02515-2 (PMC9463860; doi:10.1186/s13023-022-02515-2)
Supplement: Supplementary file 1 — Additional file 1: Known genetic syndromes with heterotaxy in the phenotype. [file 13023_2022_2515_MOESM1_ESM.docx]

Additional file 1: Known genetic syndromes with HTX in the disease phenotype

| Syndrome | OMIM# | Common or distinguishing features | Gene or copy number variant | Inheritance pattern |
| --- | --- | --- | --- | --- |
| 22q11.2 Deletion Syndrome | [#188400](https://www.omim.org/entry/188400) | Hypoparathyroidism, thymic hypoplasia with T-Cell deficiency, velopalatal insufficiency | 22q11.2 deletion, *TBX1* | De novo dominant; autosomal dominant |
| Aglossia with Situs Inversus | [#612776](https://www.omim.org/entry/612776) | Hypoglossia (anterior 2/3 of tongue), micrognathia, microstomia, high-arched palate, thickened alveolar ridge, significant respiratory distress from upper airway obstruction, normal cognitive development | None conclusively identified |  |
| Agnathia-Otocephaly Complex (1) | [#202650](https://www.omim.org/entry/202650) | Mandibular hypoplasia/agenesis, cleft palate, synotia, synophthalmia, frontal proboscis, blind-ended trachea, holoprosencephaly | *OTX2, PRRX1* | De novo dominant; autosomal recessive (case report) |
| Bardet-Biedl Syndrome (2) | [#209900](https://www.omim.org/entry/209900) [#615981](https://www.omim.org/entry/615981) [#600151](https://www.omim.org/entry/600151) [#615982](https://www.omim.org/entry/615982) [#615983](https://www.omim.org/entry/615983) [#605231](https://www.omim.org/entry/605231) [#615984](https://www.omim.org/entry/615984) [#615985](https://www.omim.org/entry/615985) [#615986](https://www.omim.org/entry/615986) [#615987](https://www.omim.org/entry/615987) [#615988](https://www.omim.org/entry/615988) [#615989](https://www.omim.org/entry/615989) [#615990](https://www.omim.org/entry/615990) [#615991](https://www.omim.org/entry/615991) [#615992](https://www.omim.org/entry/615992) [#615993](https://www.omim.org/entry/615993) [#614994](https://www.omim.org/entry/614994) [#614995](https://www.omim.org/entry/614995) [#615996](https://www.omim.org/entry/615996) [#619471](https://www.omim.org/entry/619471) [#617406](https://www.omim.org/entry/617406) [#617119](https://www.omim.org/entry/617119) | Central obesity, postaxial polydactyly, intellectual disability, hypogonadism | *ARL6, BBS1, BBS2, BBS4, BBS5, BBS7, BBS10, BBS12, BBIP1, CCDC28B, CEP290, CFAP418, IFT27, IFT74, IFT172, LZTFL1, MKKS, MKS1, PTHB1, SDCCAG8, TMEM67, TRIM32, TTC8, WDPCP* | Autosomal recessive; digenic recessive |
| Cardiac Urogenital Syndrome (3) | [#618280](https://www.omim.org/entry/618280) | Thymic involution, tracheal stenosis/narrowing, congenital diaphragmatic hernia, genital anomalies, thyroid fibrosis | *MYRF* | De novo dominant |
| Cardiofacioneurodev-elopmental Syndrome (4) | [#619123](https://www.omim.org/entry/619123) | Microcephaly, intellectual disability, hypoplastic cerebellum, nail agenesis, digital clubbing, cleft palate, multiple variable facial anomalies | *CCDC32* | Autosomal recessive |
| Carpenter Syndrome (5) | [#201000](https://www.omim.org/entry/201000) [#614976](https://www.omim.org/entry/614976) | Craniosynostosis, obesity (including increased birth weight), diaphragmatic eventration, syndactyly, polydactyly, hearing loss, short/webbed neck, pectus excavatum, pectus carinatum | *MEGF8, RAB23* | Autosomal recessive |
| DK Phocomelia Syndrome | [223340](https://www.omim.org/entry/223340) | Phocomelia, thrombocytopenia, encephalocele, urogenital anomalies | None conclusively identified | Autosomal recessive (based on clinical observation) |
| Ellis-Van Creveld Syndrome (6) | [#225500](https://www.omim.org/entry/225500) | Micromelia, neonatal teeth, short and poorly developed ribs, polydactyly, nail dysplasia | *EVC1, EVC2* | Autosomal recessive |
| Galactosialidosis | [#256540](https://www.omim.org/entry/256540) | Widespread hemangiomas, coarse facial features, corneal clouding, macular cherry red spot, conjunctival telangiectasia, seizures, dysostostis multiplex, | *CTSA* | Autosomal recessive |
| Hennekam Lymphangiectasia-Lymphedema Syndrome (7) | [#235510](https://www.omim.org/entry/235510) [#616006](https://www.omim.org/entry/616006) [#618154](https://www.omim.org/entry/618154) | Systemic lymphangiectasia, syndactyly, camptodactyly, coronal craniosynostosis, renal anomalies, protein losing enteropathy, rectal prolapse, gingival hypertrophy | *ADAMTS3, CCBE1, FAT4* | Autosomal recessive |
| Johanssen-Blizzard Syndrome | [#243800](https://www.omim.org/entry/243800) | Short stature, café au-lait macules, hypocalcemia, exocrine pancreative dysfunction with malabsorption, hypoplastic nasal alae | *UBR1* | Autosomal recessive |
| Marden-Walker Syndrome | [#248700](https://www.omim.org/entry/248700) | Growth deficiency, fixed facial expression, blepharophimosis, micrognathia, cleft palate, hypoplastic lungs | *PIEZO2* | De novo dominant, autosomal recessive (case report) |
| Nephronophthisis | [#256100](https://www.omim.org/entry/256100) [#602088](https://www.omim.org/entry/602088) [#604387](https://www.omim.org/entry/604387) [#606966](https://www.omim.org/entry/606966) [#611498](https://www.omim.org/entry/611498) [#613550](https://www.omim.org/entry/613550) [#613820](https://www.omim.org/entry/613820) [#614377](https://www.omim.org/entry/614377) [#614844](https://www.omim.org/entry/614844) [#614845](https://www.omim.org/entry/614845) [#615382](https://www.omim.org/entry/615382) [#615862](https://www.omim.org/entry/615862) [#616217](https://www.omim.org/entry/616217) [#617271](https://www.omim.org/entry/617271) | Nephronophthisis, hyposthenuria, renal failure, hepatic fibrosis | *ANKS6, CEP83, CEP164, DCDC2, GLIS2, INVS, MAPKBP1, NEK8, NPHP1, NPHP3, NPHP4, TMEM67, TTC21B, WDR19, ZNF423* |  |
| Oculo-Auriculo-Vertebral Spectrum Disorder | [#164210](https://www.omim.org/entry/164210) | Hemifacial microsomia, preauricular tags, epibulbar dermoids, blepharophimosis, microphthalmia, cleft lip/palate, parotid agenesis, vertebral anomalies | None conclusively identified | Autosomal dominant (based on clinical observation) |
| Polycystic Kidney Disease | [#173900](https://www.omim.org/entry/173900) [#613095](https://www.omim.org/entry/613095) | Polycystic kidneys, intracranial aneurysm | *PKD1, PKD2* | Autosomal dominant |
| Primary Ciliary Dyskinesia | [#244400](https://www.omim.org/entry/244400) | Chronic oto-sino-pulmonary disease, bronchiectasis, infertility | Over 45 genes identified. See OMIM [#244400](https://www.omim.org/entry/244400) | Mainly autosomal recessive |
| Renal-Hepatic-Pancreatic Dysplasia | [#208540](https://www.omim.org/entry/208540) [#615415](https://www.omim.org/entry/615415) | Polycystic liver, hepatic fibrosis, biliary cirrhosis, polycystic kidneys, ureteral atresia, cerebral cysts, potter sequence | *NEK8, NPHP3* | Autosomal recessive |
| Sandestig-Stefanova Syndrome | [#618804](https://www.omim.org/entry/618804) | Growth deficiency, progressive microcephaly, congenital cataract, optic atrophy, cleft lip/palate, pulmonary capillary hemangiomatosis, contractures, seizures | *NUP188* | Autosomal recessive |
| Short Rib Thoracic Dysplasia 3 | [#613091](https://www.omim.org/entry/613091) | Shortened ribs, micromelia, bifid tongue, lingual hamartoma, cleft lip/palate, biliary dysplasia, ambiguous genitalia, polycystic kidneys | *DYNC2H1* | Autosomal recessive; digenic recessive |

**REFERENCES**

1. Dubucs C, Chassaing N, Sergi C, Aubert-Mucca M, Attié-Bitach T, Lacombe D, et al. Re-focusing on Agnathia-Otocephaly complex. Clin Oral Investig. 2021;25(3):1353-62.

2. Olson AJ, Krentz AD, Finta KM, Okorie UC, Haws RM. Thoraco-Abdominal Abnormalities in Bardet-Biedl Syndrome: Situs Inversus and Heterotaxy. J Pediatr. 2019;204:31-7.

3. Pinz H, Pyle LC, Li D, Izumi K, Skraban C, Tarpinian J, et al. De novo variants in Myelin regulatory factor (MYRF) as candidates of a new syndrome of cardiac and urogenital anomalies. Am J Med Genet A. 2018;176(4):969-72.

4. Harel T, Griffin JN, Arbogast T, Monroe TO, Palombo F, Martinelli M, et al. Loss of function mutations in CCDC32 cause a congenital syndrome characterized by craniofacial, cardiac and neurodevelopmental anomalies. Hum Mol Genet. 2020;29(9):1489-97.

5. Victorine AS, Weida J, Hines KA, Robinson B, Torres-Martinez W, Weaver DD. Prenatal diagnosis of Carpenter syndrome: looking beyond craniosynostosis and polysyndactyly. Am J Med Genet A. 2014;164A(3):820-3.

6. Hills CB, Kochilas L, Schimmenti LA, Moller JH. Ellis-van Creveld syndrome and congenital heart defects: presentation of an additional 32 cases. Pediatr Cardiol. 2011;32(7):977-82.

7. Ivanovski I, Akbaroghli S, Pollazzon M, Gelmini C, Caraffi SG, Mansouri M, et al. Van Maldergem syndrome and Hennekam syndrome: Further delineation of allelic phenotypes. Am J Med Genet A. 2018;176(5):1166-74.
